# Public Interest in Knee Pain and Knee Replacement during the SARS-CoV-2 Pandemic in Western Europe

**DOI:** 10.3390/jcm10051067

**Published:** 2021-03-04

**Authors:** Arne Kienzle, Lara Biedermann, Evgeniya Babeyko, Stephanie Kirschbaum, Georg Duda, Carsten Perka, Clemens Gwinner

**Affiliations:** 1 Center for Musculoskeletal Surgery, Charité–Universitätsmedizin Berlin, Corporate Member of Freie Universität Berlin, Humboldt-Universität zu Berlin, and Berlin Institute of Health, 10117 Berlin, Germany; lara.biedermann@charite.de (L.B.); evgeniya.babeyko@charite.de (E.B.); stephanie.kirschbaum@charite.de (S.K.); carsten.perka@charite.de (C.P.); clemens.gwinner@charite.de (C.G.); 2 Laboratory of Adaptive and Regenerative Biology, Brigham & Women’s Hospital, Harvard Medical School, Boston, MA 02115, USA; 3 Julius Wolff Institute and Center for Musculoskeletal Surgery, Charité—Universitätsmedizin Berlin, Corporate Member of Freie Universität Berlin, Humboldt-Universität zu Berlin, and Berlin Institute of Health, 13353 Berlin, Germany; georg.duda@charite.de

**Keywords:** public interest, SARS-CoV-2, corona virus, knee osteoarthritis, total knee arthroplasty, knee pain, Google Trends

## Abstract

Due to the severe acute respiratory syndrome coronavirus type 2 (SARS-CoV-2) pandemic, a large number of elective knee replacement procedures had to be postponed in both early and late 2020 in most western countries including Germany and the UK. It is unknown how public interest and demand for total knee arthroplasties was affected. Public interest in knee pain, knee osteoarthritis and knee arthroplasty in Germany and the UK was investigated using Google Trend Analysis. In addition, we monitored for changes in patient composition in our outpatient department. As of early March in Germany and of late March in the UK, until the lockdown measures, a 50 to 60% decrease in relative search frequency was observed in all categories investigated compared to the beginning of the year. While public interest for knee pain rapidly recovered, decreased interest for knee osteoarthritis and replacement lasted until the easing of measures. Shortly prior to and during the first lockdown mean search frequency for knee replacement was significantly decreased from 39.7% and 36.6 to 26.9% in Germany and from 47.7% and 50.9 to 23.7% in the UK (Germany: *p* = 0.022 prior to lockdown, *p* < 0.001 during lockdown; UK: *p* < 0.0001 prior to and during lockdown). In contrast, mean search frequencies did not differ significantly from each other for any of the investigated time frames during the second half of 2020 in both countries. Similarly, during the first lockdown, the proportion of patients presenting themselves to receive primary knee arthroplasty compared to patients that had already undergone knee replacement declined markedly from 64.7% to 46.9%. In contrast, patient composition changed only marginally during the lockdown measures in late 2020 in both Germany and the UK. We observed a high level of public interest in knee arthroplasty despite the ongoing pandemic. The absence of a lasting decline in interest in primary knee arthroplasty suggests that sufficient symptom reduction cannot be achieved without surgical care for a substantial number of patients.

## 1. Introduction

The first verified SARS-CoV-2 infection was reported on 28 January 2020 in Germany and on 31 January 2020 in the UK. In the wake of the continuous spread of the virus, both Germany and the UK experienced the first wave of cases of the currently ongoing pandemic. At the beginning of spring, the number of new infections rapidly declined, but since October, case numbers have risen sharply again in both countries [1].

A large number of preventive measurements were devised and implemented over the course of 2020. To avert an imminent overburdening of hospitals, a “lockdown” was imposed in March by both the German and UK government. In addition to shutting down a large number of recreational facilities, officials urged the public to minimize social interactions in an effort to prevent the spread of SARS-CoV-2 [2]. Concurrently, clinics were instructed to significantly limit the number of elective surgeries, including total knee joint arthroplasty [3,4]. With the easing of restrictions over the spring in Germany and later in summer in the United Kingdom, total knee arthroplasty surgery was available again for knee osteoarthritis patients. However, the renewed rise in SARS-CoV-2 case numbers in the fall led to a significantly less restrictive “lockdown light” in October in Germany [5]. Unlike the first lockdown, a variety of sports and recreational facilities remained operational. In addition, social interactions were limited, but not to the same extent as during the first lockdown in March. In December, declining ICU bed capacities led to the political decision of a second lockdown with a renewed directive to significantly reduce elective surgeries [6]. In contrast, the British government opted for a second lockdown in November that only lasted for approximately one month [7].

The implemented reduction in elective procedures competes with the oftentimes severe level of symptoms experienced by patients with knee osteoarthritis [8,9]. A considerable number of patients with advanced knee osteoarthritis report progressive severe pain and substantially limited mobility over an extended period of time [8,10,11].

It is unknown how public interest and need for medical consultation in regard to knee osteoarthritis and knee replacement was affected during the first and second wave of the SARS-CoV-2 pandemic in Europe. The present study explores the potential impact of SARS-CoV-2 caseloads and resulting government policies on public interest in knee replacement using Google Trends and analyzing patient composition in our outpatient department.

## 2. Materials and Methods

### 2.1. Google Trend Analysis

Public interest was investigated using Google Trend analysis as previously described [12]. In short, daily relative search frequencies for knee pain, knee osteoarthritis and knee replacement were assessed for the entire year 2020 in Germany and the UK. 7-day floating averages of relative search frequencies and new SARS-CoV-2 infections were calculated and plotted over time. Key political decisions and events were annotated in the regarding graphs. Time frames used were: T1_GER (1 January–27 January), T2_GER (28 January–1 March), T3_GER (2 March–22 March), T4_GER (23 March–26 April), T5_GER (27 April–30 June), T6_GER (1 July–1 November), T7_GER (2 November–16 December), T8_GER (17 December–31 December); T1_UK (1 January–30 January), T2_UK (31 January–14 March), T3_UK (15 March–22 March), T4_UK (23 March–24 June), T5_UK (1 July–4 November), T6_UK (5 November–2 December), and T7_UK (3 December–31 December). Relative search frequency for knee arthroplasty were averaged for the defined time periods to compare for significant changes. Significance was tested for using Mann–Whitney *U* test.

### 2.2. SARS-CoV-2 Case Numbers

The absolute number of new SARS-CoV-2 infections was obtained from the official website of the Robert Koch Institute for Germany and from the official “Coronavirus” website of the UK government on 5 January 2021 [1,13]. Case numbers were calculated and graphed as 7-day floating averages.

### 2.3. Outpatient Department

Patient volume and composition were analyzed for our outpatient department. Patients were categorized into two groups by reason for referral to our hospital: Patients with knee osteoarthritis to assess clinical indications for primary knee replacement versus patients that have already undergone knee arthroplasty.

## 3. Results

### 3.1. Public Interest

**Figure 1:**
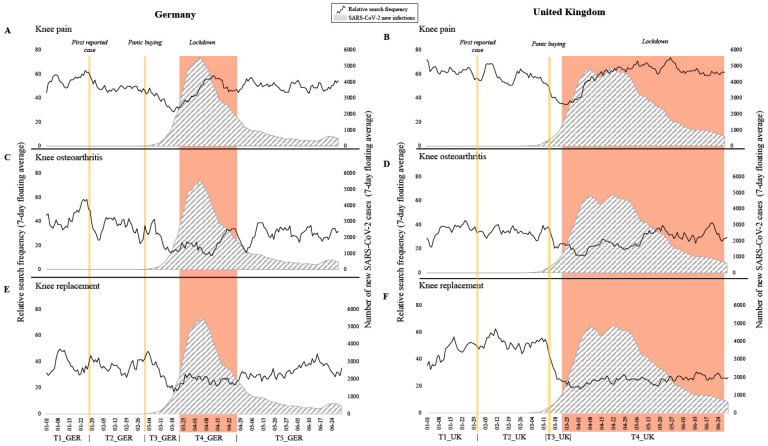
Public interest as relative search frequencies (black curve) of (**A**,**B**) knee pain, (**C**,**D**) knee osteoarthritis, and (**E**,**F**) knee prosthesis and number of new SARS-CoV-2 infections (grey, striped area) in the first half of 2020 in Germany and the UK, respectively. Relevant events and time frames are highlighted in color.

In the beginning of the year, relative search frequencies fluctuated by ±20 points for any of the three investigated search terms until the first detected SARS-CoV-2 infection was reported in Germany and the UK (Figure 1; T1_GER and T1_UK). Following the first reported case in Germany, a discrete decline in interest in knee pain and knee osteoarthritis can be noted (T2_GER). In contrast, no difference was observed in interest in knee replacements in Germany during this time period. Similarly, interest did not decline for any of the investigated search queries in the UK until first panic buying occurred (T2_UK). As of early March, until the lockdown measures, a 50 to 60% decrease in relative search frequency was observed in all categories investigated compared to the beginning of the year in Germany (T3_GER). A similar trend can be observed in the UK starting from late March (T3_UK). Besides increasing infection rates in Germany and the UK, numerous media reports on high rates of new SARS-CoV-2 infections in Italy and on panic buying were published. Accompanying the first lockdown (T4_GER and T4_UK) is a recovery of public interest in knee pain, whereas interest in knee osteoarthritis and knee replacement initially remained low in both countries at first. Lockdown measurements in the UK lasted approximately two months longer than in Germany. In the UK, interest in knee osteoarthritis and knee replacement recovered only slightly towards the end of the measures. Following the easing of restrictions, interest in knee osteoarthritis and knee prosthesis surged to pre-pandemic levels in Germany (T5_GER).

**Figure 2:**
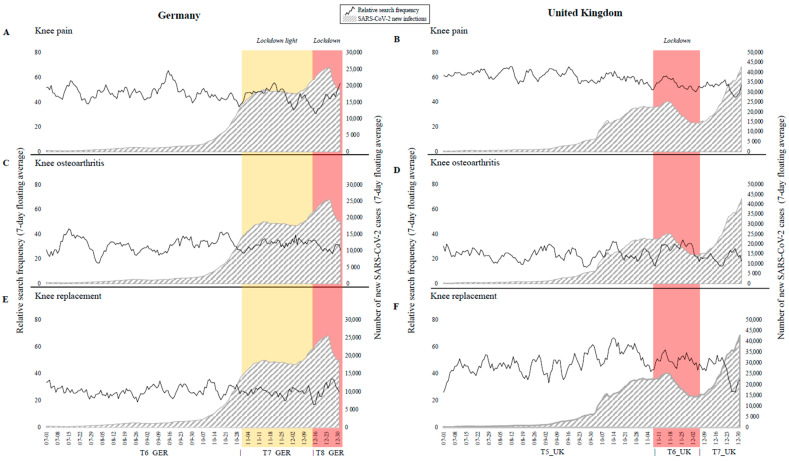
Public interest as relative search frequencies (black curve) of (**A**,**B**) knee pain, (**C**,**D**) knee osteoarthritis, and (**E**,**F**) knee prosthesis and number of new SARS-CoV-2 infections (grey, striped area) in the second half of 2020 in Germany and the UK, respectively. Relevant times frames are highlighted in color.

In contrast to the data from the first half year, no declining interest in the investigated topics was observed in the third and fourth quarter of 2020 (Figure 2). Neither the rising case numbers from October onwards nor the lockdown light (T7_GER) showed an impact on search behavior in Germany. Similarly, the second lockdown had little to no impact on public interest on any of the investigated search queries both in Germany (T8_GER) and the UK (T6_UK). Search frequency for knee osteoarthritis and knee replacement declined in both countries in the last two weeks of the year.

**Figure 3:**
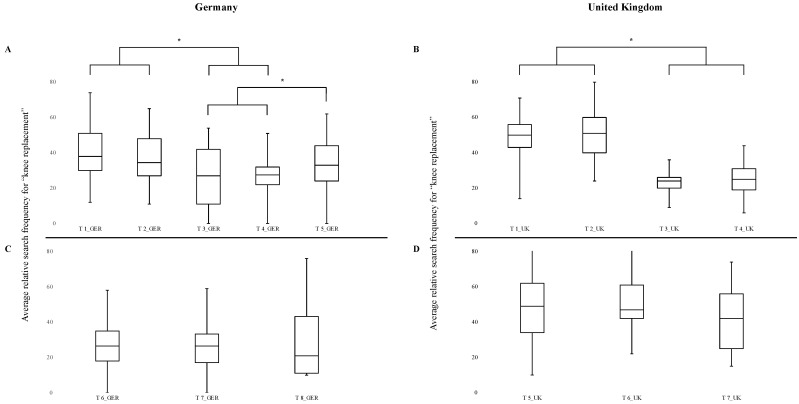
Comparison of mean search frequencies for knee arthroplasty. (**A**,**B**) First half of 2020. Mean search frequencies were calculated from January 1 till the first confirmed SARS-CoV-2 case (T1_GER and T1_UK), till the first panic buying (T2_GER and T2_UK), till the lockdown (T3_GER and T3_UK), till the end of the lockdown (T4_GER and T4_UK), and till the end of the 2nd half of the year (T5_GER). (**C**,**D**) Second half of 2020. Mean search frequencies were calculated till the start of the lockdown light in Germany (T6_GER), till the start of the second lockdown (T7_GER and T5_UK), and till the end of 2020 (T8_GER and T6_UK). * *p* < 0.05.

Mean search frequencies for knee arthroplasty showed no significant difference for the time period after the first detected SARS-CoV-2 case (T2_GER: 36.6% and T2_UK: 50.9%) compared with pre-pandemic values (T1_GER: 39.7% and T1_UK: 50.9%; Figure 3A,B). When first reports about panic buying emerged (T3_GER: 26.9% and T3_UK: 23.7%), a significant decrease in public interest compared to T1_GER (*p* = 0.022), T1_UK (*p* < 0.0001), T2_GER (*p* = 0.037), and T2_UK (*p* < 0.0001) can be noted. Public interest remained significantly lower during lockdown measurements in Germany (T4_GER: 26.5%) and the UK (T4_UK: 24.9%) compared to T1_GER (*p* < 0.001), T1_UK (*p* < 0.0001), T2_GER (*p* < 0.001), and T2_UK (*p* < 0.0001). No significant difference was found in public interest after the easing of lockdown measurements in Germany (T5_GER: 33.4%). In contrast, mean search frequencies did not differ significantly from each other for any of the investigated time frames during the second half of 2020 (T6_GER: 27.1%, T7_GER: 25.1, and T8_GER: 30.0%; T6_UK: 48.9%, T7_UK: 50.1%, and T8_UK: 41.9%; Figure 3C,D).

### 3.2. Patient Composition in Outpatient Department

**Figure 4:**
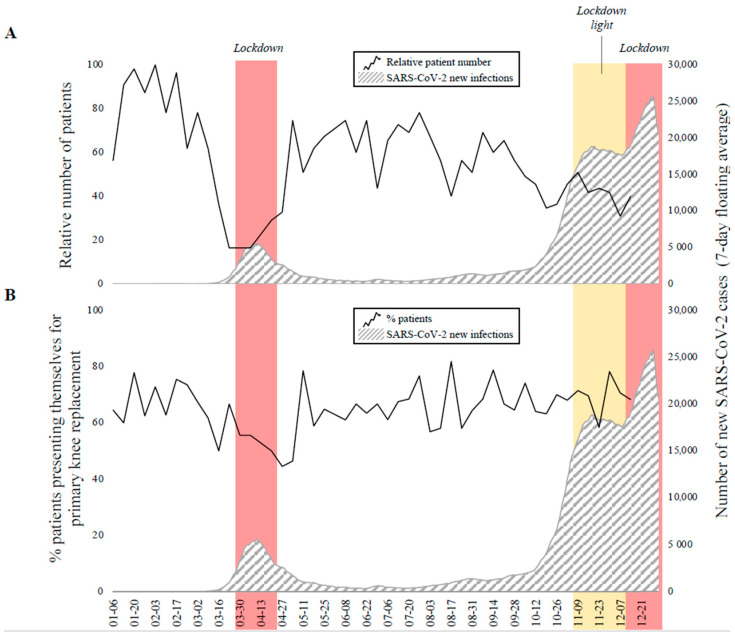
(**A**) Relative patient numbers (black curve) referred to our outpatient department and number of new SARS-CoV-2 infections (grey, striped area). (**B**) Percentage of patients with knee osteoarthritis to assess clinical indications for primary knee replacement in our outpatient department.

During the lockdown in March 2020 and the lockdown light followed by the second lockdown in December 2020, the number of patients in our outpatient department fell by up to 80% (Figure 4A). After easing of the measures in spring, the number of patients presenting themselves quickly recovered to previous levels. Similarly, during the first lockdown, the proportion of patients presenting themselves to receive primary knee arthroplasty compared to patients that had already undergone knee replacement declined markedly from 64.7 to 46.9% (Figure 4B). In contrast, patient composition changed only marginally during the lockdown light (69.2% patients requesting primary knee replacement) in December. Due to the timing of the second lockdown coinciding with the Christmas holidays, no analysis of outpatient department composition could be performed for this time period.

## 4. Discussion

In this study, we demonstrated a significant decline in public interest in knee osteoarthritis and knee arthroplasty as a result of the policies implemented during the first lockdown both in Germany and the UK. In contrast, no such decline was observed during the lockdown light in Germany and second lockdown in both countries. The analyzed Google Trends data correlated with patient composition in our outpatient department.

We hypothesize that the decreased public interest as well as the reduced number of patient presentations for primary knee arthroplasty is due to a pandemic-specific reprioritization of patients’ needs. The majority of patients presenting themselves in our outpatient department with advanced knee osteoarthritis report progressive severe pain and substantially limited mobility over an extended period of time [8,10,11]. Presumably, the fear of contracting SARS-CoV-2 and of an impending collapse of the health care system had resulted in a temporary reprioritization of pain in a significant number of patients [14,15].

Interestingly, a dip in public interest was evident in both the Google Trends analysis and in our outpatient department patient composition even before the first lockdown. Upon the first surge of confirmed SARS-CoV-2 cases in Germany and the UK and the ensuing public uncertainty, which was reflected in panic buying, patients appeared to reprioritize their medical needs. Previous studies have demonstrated the medical relevance of Google Trends analytics in the spread of influenza in the U.S.-similar to our findings, changes in public interest were evident prior to increasing case numbers and hospitalizations [16,17]. Likewise, Landy et al. were able to describe a decline in public interest in knee replacements in the U.S. using Google Trends during the first wave of SARS-CoV-2 infections [12]. However, in this study it remained unclear whether this decrease reflected in changes in patient behavior. In our study, a decline in public interest corresponded with changed patient composition in our outpatient department-prior to and during the first lockdown, the number of patients presenting themselves for primary total knee arthroplasty was significantly decreased.

Conversely, the increased percentage of patients presenting themselves due to medical issues after knee arthroplasty suggests that this group of patients was not able to reprioritize their needs due to the severe symptom burden [18,19]. These observations suggest that Google Trend analysis can be used as an adequate predictor of changing patient needs in orthopedics.

While public interest in knee pain rebounded to pre-lockdown levels within weeks, this was not the case for knee osteoarthritis and knee replacement. We speculate that the rapid recovery in interest in knee pain after the first lockdown is due to the fact that, unlike in osteoarthritis, younger patients are more likely to be affected. An increase in physical activities, especially running, during the first lockdown potentially bolsters this trend [20,21]. In addition, the high level of confidence in political institutions and healthcare system in Germany compared to the United Kingdom may have contributed to the faster recovery of public interest in Germany [22,23].

In contrast to our observations during the first lockdown, there was no significant decline in public interest or patient composition during the lockdown measures in the second half of 2020. We postulate that the prospect of a prolonged lockdown coupled with the high disease burden in patients with knee osteoarthritis did not result in the same reprioritization as during the first lockdown. In line with these findings, in a study conducted in the USA during the first lockdown, the majority of patients with knee osteoarthritis indicated that they would seek surgical treatment as soon as possible after measures are eased [24]. Concurrently, it remains debatable whether our observations corroborate claims that the lockdown measures in late 2020 were not taken seriously enough by a significant portion of the population to effectively reduce the SARS CoV-2 caseload [25]. Our data suggests a shift in public perception of the enacted pandemic measures over the year. It must be critically discussed whether the Christmas holidays and other political events confound with effects of the coinciding lockdown measures at the end of the year.

Despite consistently high disease burden in a significant number of the osteoarthritis patients, hospitals had to reduce surgical capacities to ensure provision of care for SARS-CoV-2 patients. The data presented in this report illustrate a sustained, high level of public interest in knee arthroplasty despite the ongoing pandemic. Utilization of conservative treatment options and strict patient triaging based on the severity of symptoms could potentially mitigate the impact of the necessary lockdown measures on knee osteoarthritis patients [26,27]. However, the absence of a lasting decline in interest in primary knee arthroplasty suggests that sufficient symptom reduction cannot be achieved without surgical care for a substantial number of patients.

## Data Availability

All Google Trends data presented in this study are openly available at www.trends.google.com. The remainder of the data presented in this study are available on request from the corresponding author.
